# Data on growth performance, glucose concentration and testosterone level of Asian seabass, *Lates calcarifer* juveniles fed with exogenous melatonin at different concentration

**DOI:** 10.1016/j.dib.2022.108495

**Published:** 2022-07-30

**Authors:** Nur Farihah Rani, Muhamad Yazed Abduh, Nor Hakim Norazmi-Lokman, Siti Ariza Aripin

**Affiliations:** a Faculty of Fisheries and Food Science, Universiti Malaysia Terengganu, Kuala Terengganu, Terengganu 21030, Malaysia; b Centre for Fisheries and Aquaculture, Institute for Marine and Antarctic Studies, University of Tasmania, Taroona, Tasmania 7053, Australia

**Keywords:** Aquaculture, Tropical fish, Barramundi, Fish feed, Hormone administration

## Abstract

This data article describes the growth performance, glucose concentration in blood and testosterone level in plasma of juvenile Asian seabass (*Lates calcarifer*) after being fed with exogenous melatonin at different concentrations (0, 50 and 100 mg kg^−1^). To collect the data, 160 juveniles (60 days old) with an initial mean weight of 20.54 ± 7.16 g and mean length 11.14 ± 0.05 cm were reared in 1800 L rectangular fibreglass aerated tanks of a recirculating aquaculture system (*n* = 40 fish/ tank/ treatment) for 90 days. Four different treatments were tested: basal diet without any hormone (Control), basal diet with ethanol solution (Control + Ethanol; vehicle control), basal diet sprayed with 50 mg/kg feed of melatonin (Melatonin (50 mg/kg of diets)) and basal diet sprayed with 100 mg/kg feed of melatonin (Melatonin 100 mg/kg of diets). Initial and final body weight and body length of fish was measured and recorded to calculate the data of weight gain (WG). At the end of the feeding trial, the juveniles blood glucose and testosterone level were analyzed. Normality test, One-way ANOVA analysis followed by Tukey post-hoc test were then performed on the data obtained from the calculation of WG, survival rate, blood glucose and testosterone level. The data presented in this article will helps farmers and scientist to optimize the usage of melatonin administration in Asian seabass juveniles.

## Specifications Table

**Table:** 

Subject	Aquatic Science
Specific subject area	Aquaculture
Type of data	Table Graph
How the data were acquired	Data were obtained by physical measurements such as calculation of growth rate, gonadosomatic index and hepatosomatic index. Chemical parameters such as glucose concentration were measured at the end of feeding trial by using a portable glucometer. Data on testosterone level were collected and analyzed using ELISA analysis. Statistical analysis was performed using IBM SPSS software version 25 (IBM, US).
Data format	Raw Analyzed
Description of data collection	For weight gain, measurement of body weight and total length of the fish were done at the initial (day 0) and final of the feeding trial (day 90). For survivability, the number of live fish was counted at the end of the experiment. For glucose level, the blood was withdrawn from fish and analyzed using a portable glucometer: Accu-Chek Glucotrend 2 (Roche Diagnostics, Germany). For testosterone level, the blood plasma was analyzed using ELISA kit Testosterone (Cayman Chemical Company) and the procedure was done according to the protocol of the kit.
Data source location	• Institution: Faculty of Fisheries and Food Sciences • City/Town/Region: Kuala Terengganu, Terengganu • Country: Malaysia • Latitude and longitude (and GPS coordinates, if possible) for collecting samples/data: Freshwater Hatchery, Faculty of Fisheries and Food Sciences, Universiti Malaysia Terengganu: 5 °24′ 36.2″ N 103 °05′ 20.2″ E
Data accessibility	1. With the article 2. Repository name: Mendeley Data Data identification number: DOI: 10.17632/bnh7myhsbv.1 Direct URL to data: https://data.mendeley.com/datasets/bnh7myhsbv/1

## Value of the Data

• The application of melatonin in tropical fish feed as an enhancer (growth and reproduction) has not been widely used. This dataset will help scientists and farmers to fully explored melatonin application and potential in the industry.
• These data will benefit aquaculture or fish biology researchers and tropical fish farmers.
• These data can be use by farmers and scientists to optimize the application of melatonin in fish feed thus increasing the production of this species.

## 1 Data Description

**Fig. 1:**
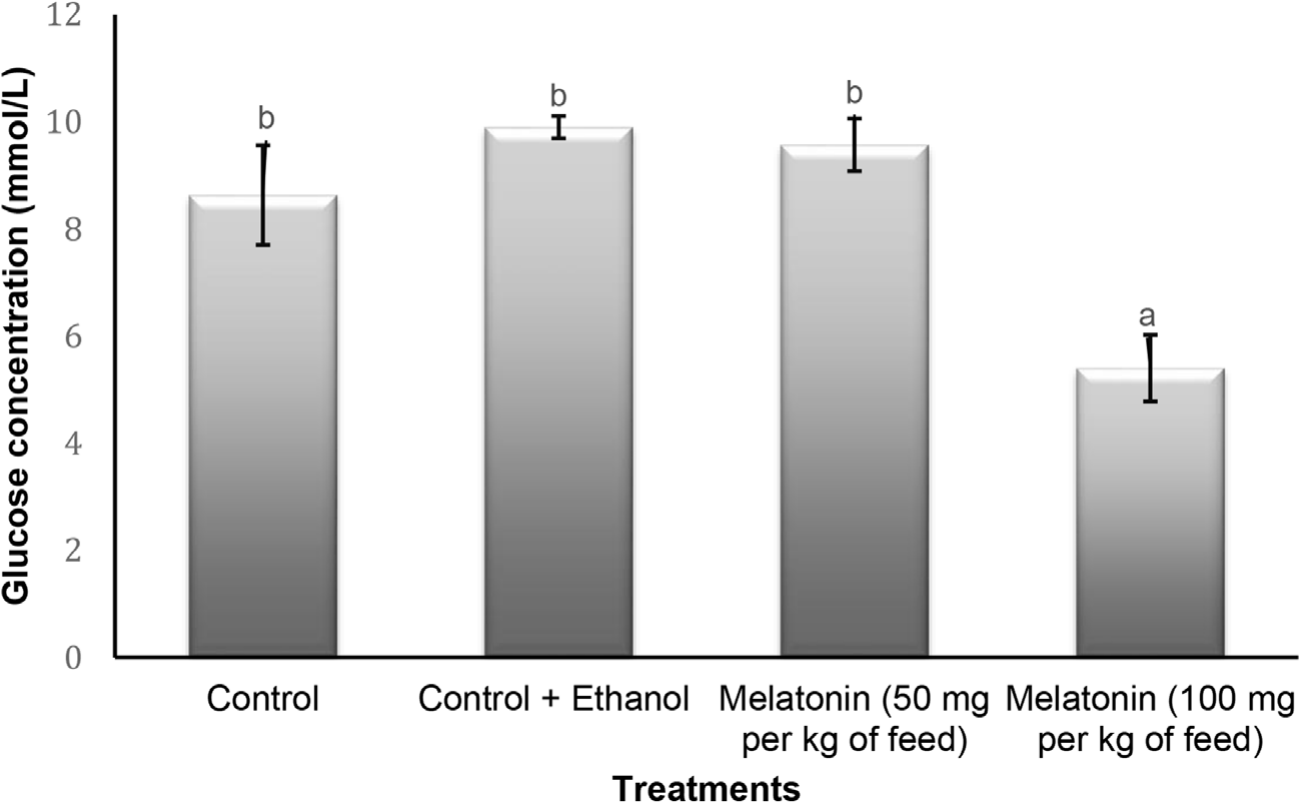
Glucose concentration in blood of *Lates calcarifer* juveniles after 90 days administration of exogenous of melatonin at different concentration. Values are mean with standard error. Different letters within each series indicate significant (*p* < 0.05) difference.

**Fig. 2:**
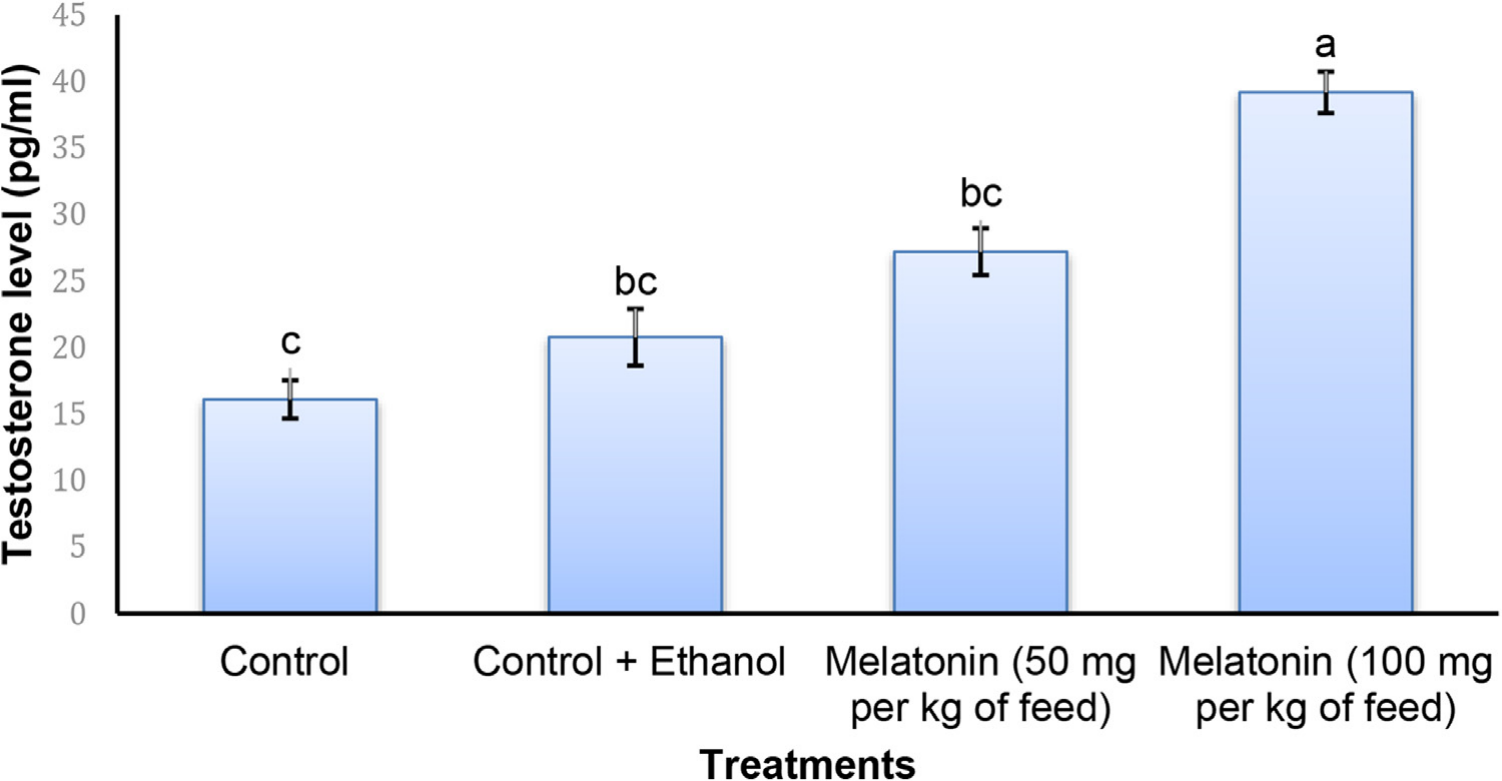
Testosterone level in plasmaof *Lates calcarifer* juveniles after 90 days administration of exogenous of melatonin at different concentration. Values are mean with standard error. Different letters within each series indicate significant (*p* < 0.05) difference.

The raw data on growth performance, glucose concentration and testosterone level of Asian seabass, *Lates calcarifer* juveniles fed with exogenous melatonin at different concentration are as in [1]. Table 1 describes the weight gain (WG), survival rate, GSI and HSI level of the seabass after the experimental period. Meanwhile, glucose concentration in blood is presented in Fig. 1 and the testosterone level in plasma is presented in Fig. 2. All data in the table and figures is presented as mean ± standard error.

**Table 1:** Growth performance of Asian seabass, *Lates calcarifer* after fed with melatonin at different concentration. Values are means ± standard error. Different letters used in each row indicate the significant (*p* < 0.05) difference.

Growth parameter	Control	Control + Ethanol	Melatonin (50 mg/kg of diets	Melatonin (100 mg/kg of diets	*p*- value
Initial mean body weight (g)	20.03 ± 0.33	21.18 ± 0.46	20.53 ± 0.75	20.43 ± 0.43	0.47
Initial mean body length (cm)	11.21 ± 0.07	10.97 ± 0.07	11.29 ± 0.14	11.10 ± 0.12	0.15
Final mean body weight (g)	52.13 ± 1.74^c^	56.99 ± 1.62^c^	75.83 ± 3.63^b^	87.16 ± 4.90^a^	0.01
Final mean body length (cm)	15.46 ± 0.10^d^	18.12 ± 0.13^c^	19.37 ± 0.26^b^	20.21 ± 0.47^a^	0.01
Weight gain (g)	482.43	538.23	839.0	993.2	-
Survival rate (%)	90	90	92.5	95	-
GSI (%)	0.39 ± 0.04^b^	0.22 ± 0.03^b^	0.49 ± 0.07^a^	0.26 ± 0.18^b^	0.01
HSI %)	1.55 ± 0.20	1.39 ± 0.09	1.21 ± 0.34	1.51 ± 0.38	0.83

## 2 Experimental Design, Materials and Methods

### 2.1 *Lates calcarifer* Juveniles

Juveniles *Lates calcarifer* were obtained from a local hatchery in Banting, Selangor and were adapted to the experimental condition in Freshwater Hatchery of the Faculty of Fisheries and Food Science, Universiti Malaysia Terengganu, Malaysia. All fish were placed and acclimatized in 1800 L fibre glass aerated tanks of a recirculating aquaculture system. The fish were fed with the control diet to satiation during this acclimatization period. After two weeks, a total of 160 juveniles of Asian seabass with an initial mean weight 20.54 ± 7.16 g (mean ± SE) and length 11.14 ± 0.05 cm in length was randomly selected and divided into four treatments (*n* = 40 per treatment/tank; 20 L/fish). They were initially starved for two days and subjected to normal photoperiod (12 h daylight) and temperature (28–30 °C) prior to the feeding trial. The water quality was monitored daily and maintained at salinity 0 ppt; pH 7.4–8.3; dissolved oxygen 6.0–7.3 ppm and total ammonia nitrogen 0.08–0.11 mg L^-1^ using water test kits and dissolved oxygen meter.

### 2.2 Control and Melatonin Containing Diet Preparation

Following the requirements for optimum growth of juvenile Asian seabass, the basal diets were formulated based on [2] with modifications and proximate composition of the diets as described in (Table 2). All the dry ingredients were mixed together followed by addition of oil and water. The mixture dough was then pelleted using a pasta maker using a 3 mm die. Then, the control diets were dried for 24 h at 60 °C in the oven. In addition to the control diet, other three experimental diets were prepared with the addition of ethanol solution and melatonin. The diets containing melatonin were characterized as follows (Table 3):

**Table 2:** Feed ingredients weight and proximate composition of the basal diets fed to Asian seabass, *Lates calcarifer*.

Feed Ingredients	Percentage (%)
Fish meal^a^	30
Soybean meal^a^	34
Wheat flour^a^	10.5
Rice bran^a^	5
Spirulina^a^	0.8
Tapioca	9.6
Soy lecithin^a^	1.5
Calcium phosphate^a^	1.3
Magnesium sulphate^a^	0.1
Potassium chloride^a^	0.5
CMC^a^	2
Vitamin premix^a^^,^^b^	0.5
Mineral premix^a^^,^^c^	0.5
Fish oil^a^	1.5
Soya oil^a^	2.2
Proximate composition (%)	
Protein	43.76 ± 1.40
Lipid	8.57 ± 0.41
Fibre	1.9 ± 0.10
Ash	10.45 ± 0.14
Moisture	9.61 ± 0.09

a Sri Purta Trading, Alor Setar, Kedah.

b Vitamin premix (mg kg^−1^): Thiamine-HCl, 8.0; Riboflavin, 8.0; Niacinmix, 100.0; Pyridoxine-HCl, 20.0; Cyanocobalamine, 0.1; Pantothenate, 20.0; Biotin, 1.0; Inositol, 100.0; Folic acid, 5.0; Ascorbic acid, 250.0; Vitamin A, 20.0; Vitamin D, 8.0; Vitamin E, 150.0; Vitamin K, 10.0; BHT,10.0; α-cellulose, 1289.9.

c Mineral premix (mg kg^−1^): MgSO_4_·_7_H_2_O, 300.0; FeSO_4_·_7_H_2_O, 180.0; ZnSO_4_·7H2O, 120.0; MnSO_4_·_7_H_2_O, 35.0; KI, 0.65; Na_2_SeO_3_, 0.5; CoCl·_6_H_2_O (1%), 7.0; CuSO_4_·_5_H_2_O, 5.0; Zeolite, 7351.85.

**Table 3:** Description of treatments.

Treatments	Description
Control	Basal diets; control diets
Control + Ethanol	Basal diets + ethanol solution (vehicle control)
Melatonin (50 mg/kg of diets)	Basal diets + 50 mg of melatonin/ kg of diets
Melatonin (100 mg/kg of diets)	Basal diets + 100 mg of melatonin/ kg of diets

For Control treatment, basal diets were fed to the fish. The vehicle control treatment (Control + Ethanol) treatment was prepared by spraying 150 ml of 70% ethanol on 1 kg of feed. The melatonin-containing diets was prepared using commercial melatonin tablets (Piping Rock Health Products, USA). The two melatonin treatments was prepared by diluting 50 mg and 100 mg of melatonin, respectively, into 150 ml of 70% ethanol solution before each solutions were sprayed homogenously onto 1 kg of basal diets. The tested concentration of melatonin were selected and modified based on [2,3]. After the hormone spraying and mixing process, the diets was allowed to dry at room temperature for 24 h before being kept in an airtight black container and stored in refrigerators (4 °C) until usage to avoid contamination [3].

The feeds were administrated at the rate of 5% of the body weight and this amount of diet was divided into two equal feedings per day (0900 and 1600) then they were fed till satiation for 90 days. In order to maintain a healthy environment, uneaten food and feces were siphoned out after each feeding half an hour after each feeding session.

### 2.3 Growth Performance

Sampling was done at day 0 (initial) after the adaptation of fishes to the experimental system (acclimatization period) and at day 90 (final). They were starved for 24 h to empty the alimentary tract contents prior to sampling procedures. Then, the fish were anesthetized using clove oil (50 mg/L) [4]. The initial and final body weight and body length of each fish were measured and recorded. The survival rate and growth performance were calculated. Then, three fishes (*n* = 3) per tank were randomly selected and humanely sacrificed, where their liver and gonad were carefully removed and weighed for calculating the gonadosomatic Index (GSI) and hepatosomatic index (HSI). The following formula were used [5].

• Weight gain, WG (g) = (Final body weight- Initial body weight)
• Survival (%) = (Number of fish in each treatment remaining at the end of experimental / initial number of fish) × 100
• Gonadosomatic Index, GSI (%) = 100 (Mass of gonad/ body weight of fish)
• Hepatosomatic Index, HSI (%) = 100 (Mass of liver/ body weight of fish)

### 2.4 Glucose Test

At the end of the feeding trial, after weighing and measuring the body weight and length, three fish (*n* = 3) from each treatment were randomly selected to anaesthetize. Blood was then drawn from the caudal vein using 3 ml syringe with 21 gauge coated with Ethylenediaminetetraacetic acid (EDTA) solution to prevent clotting of blood taken. The stripes were firstly inserted into the portable Glucometer Accu-Chek Glucotrend 2 (Roche Diagnostics, Germany), then one drop of blood was dropped onto the strip before the values displayed by the glucometer in seconds [6]. Measurement of the glucose concentration was done in monoplicate due to the small volume of sample.

### 2.5 Testosterone Analysis

For testosterone analysis, five fish (*n* = 5) from each tank were sampled. Blood from five fish was then pooled for each treatment. Fish blood samples were taken using 3 ml syringe with 21 gauge coated with (EDTA) solution. One ml of blood sample was then pooled (1 ml) for each treatment and transferred each to 1.5 ml tube. The blood was allowed to separate and left to clot for 15 min at 4 °C. Afterwards, the tubes were centrifuged (5000 rpm, 4 °C, 15 min) using an Eppendorf Centrifuge to separate the the blood and plasma. The plasma was then transferred to –80 °C until the assays of hormone [7]. The testosterone level was analyzed using the ELISA kit Testosterone (Cayman Chemical Company) and the analysis of hormone content in the plasma were conducted following the protocol provided in the kit [8].

### 2.6 Statistical Analysis

All data were tested for normality and homogeneity of variance using the Shapiro-Wilks test. The experimental results were analyzed by one-way analysis of variance (ANOVA) followed by Tukey's post hoc test to statically determine the significant differences between the treatments means [9]. Differences were considered to be significant at P <0.05 by using IBM SPSS Statistics software (version 25). All analyzed data are presented as mean ± standard error.

## Ethics Statements

The authors confirm that all experiments comply with the ARRIVE guidelines and were carried out in accordance with the U.K. Animals (Scientific Procedures) Act, 1986 and associated guidelines, EU Directive 2010/63/EU for animal experiments, or the National Institutes of Health guide for the care and use of Laboratory animals (NIH Publications No. 8023, revised 1978). Moral and ethical aspect of the research such as animal handling and minimum amount of fish needed for a valid statistical analysis also complied with the Research Ethics Guidelines of Universiti Malaysia Terengganu.

## CRediT authorship contribution statement

**Nur Farihah Rani:** Methodology, Investigation, Formal analysis, Writing – original draft, Writing – review & editing. **Muhamad Yazed Abduh:** Conceptualization, Methodology, Writing – review & editing. **Nor Hakim Norazmi-Lokman:** Conceptualization, Supervision, Writing – review & editing. **Siti Ariza Aripin:** Conceptualization, Methodology, Supervision, Funding acquisition, Writing – review & editing.

## Declaration of Competing Interest

The authors declare that they have no known competing financial interests or personal relationships that could have appeared to influence the work reported in this paper.

## Data Availability

Raw Data on Growth Performance, Glucose Concentration and Testosterone Level of Asian seabass, Lates calcarifer Juveniles Fed with Exogenous Melatonin at Different Concentration (Original data) (Mendeley Data).
